# Whole-Genome Sequence Data for the Holotype Strain of Diaporthe ilicicola, a Fungus Associated with Latent Fruit Rot in Deciduous Holly

**DOI:** 10.1128/mra.00631-22

**Published:** 2022-08-22

**Authors:** Isabel B. Emanuel, Zachary M. Konkel, Kelsey L. Scott, Guillermo E. Valero David, Jason C. Slot, Francesca Peduto Hand

**Affiliations:** a Department of Plant Pathology, The Ohio State University, Columbus, Ohio, USA; b Center for Applied Plant Sciences, The Ohio State University, Columbus, Ohio, USA; University of Maryland School of Medicine

## Abstract

Diaporthe ilicicola is a newly described fungal species that is associated with latent fruit rot in deciduous holly. This announcement provides a whole-genome assembly and annotation for this plant pathogen, which will inform research on its parasitism and identification of gene clusters involved in the production of bioactive metabolites.

## ANNOUNCEMENT

*Diaporthe* species (*Phomopsis*; anamorph) are ascomycete fungi found as saprophytes, endophytes, and plant pathogens (1) and are recognized for their diverse production of metabolites (2). This report announces the whole-genome assembly and annotation of Diaporthe ilicicola, a newly described species associated with fruit rot in winterberry (*Ilex* spp.). Data will inform research to understand how plant-pathogenic *Diaporthe* species differ from those that do not cause disease and will aid in the identification of fungal gene clusters involved in bioactive metabolite production.

Diaporthe ilicicola holotype strain FPH2015-502 (CBS 144318) was isolated as described by Lin et al. (3). A single spore culture was maintained in long-term storage at −20°C. Diaporthe ilicicola FPH2015-502 was grown in potato dextrose broth for 2 days at 28°C and 150 rpm. DNA was extracted with the DNeasy Plant minikit (Qiagen) according to the manufacturer’s recommendations and sequenced with both short- and long-read sequencing. Illumina library preparation and sequencing were conducted at Novogene, Inc., using a NovaSeq 6000 system with paired-end 150-bp reads. The long-read sequencing library was prepared using the Oxford Nanopore Technologies SQK-LSK108 kit under standard preparation conditions with g-TUBE fragmentation. Long reads were sequenced using a Nanopore MinION Mk1B flow cell with R9.4.1 chemistry.

For genome assembly and annotation, default parameters were used for all software unless specified otherwise. Illumina read quality was checked using FastQC v0.11.8, and reads were trimmed using Trimmomatic v0.36 (options: HEADCROP:10 CROP:145 SLIDINGWINDOW:50:25 MINLEN:100) (4) for a total of 24,778,254 paired-end reads. Nanopore data were base called with Guppy v2.1.3 using the baseline model (Oxford Nanopore Technologies); reads were quality filtered and adapters were removed with Porechop v0.2.4 (5) for total of 136,483 reads (total bases, 0.62 Gbp; read *N*_50_, 7,501 bp) (Table 1). Hybrid genome assembly was performed using SPAdes v3.12.0 (kmer lengths, 21, 33, 55, 77, and 99) (6), FastQC v0.11.8 (7) was used to check assembly quality, and QUAST v4.6.3 (8) was used to compare the assembly with one reference organism, Diaporthe helianthi strain 7/96 (GenBank accession number MAVT00000000) (9). Repetitive elements were masked using RepeatModeler v1.0.11 (10) referencing the Dfam 3.0 repeat library (11). The assembly was annotated using the Funannotate pipeline v1.8.1 (12) with the following associated software: GlimmerHMM v3.0.4 (13), GeneMark-ES v4.35 (14), SNAP v2006-07-28 (15), BUSCO v3.0.2 (16), and AUGUSTUS v3.3.3 (17). Protein evidence was supplied from seven *Sordariomycetes* species, including Diaporthe ampelina UCDDA912 (GenBank accession number LCUC00000000) (18), D. helianthi 7/96 strain (GenBank accession number MAVT00000000) (9), Cytospora leucostoma (BioSample accession number SAMN04099706), Valsa mali (BioSample accession number SAMN03203459), Valsa malicola (BioSample accession number SAMN04099704), Valsa mali var. *pyri* (BioSample accession number SAMN03203462), and Valsa sordida (BioSample accession number SAMN04099705). The annotation was finalized using a modified version of OrthoFiller v1.2xonq (19) (https://gitlab.com/xonq/orthofiller) referencing 15 *Diaporthales* species, namely, Coniella lustricola (https://mycocosm.jgi.doe.gov/Pilidi1/Pilidi1.home.html), Cryphonectria parasitica (BioSample accession number SAMN02744051), *Cryptodiaporthe* sp. (https://mycocosm.jgi.doe.gov/Crypto1/Crypto1.home.html), Cryptodiaporthe populea (https://mycocosm.jgi.doe.gov/Crypo1/Crypo1.home.html), Cytospora chrysosperma (https://mycocosm.jgi.doe.gov/Cytch1/Cytch1.home.html), Cytospora leucostoma (BioSample accession number SAMN04099706), Diaporthe ampelina (https://mycocosm.jgi.doe.gov/Diaam1/Diaam1.home.html) (18), Diaporthe citri (BioSample accession number SAMN15772025), Diaporthe helianthi (https://mycocosm.jgi.doe.gov/Diahe1/Diahe1.home.html) (9), *Diaporthaceae* sp. (https://mycocosm.jgi.doe.gov/DiaPMI573_1/DiaPMI573_1.home.html), Lollipopaia minuta (https://mycocosm.jgi.doe.gov/Lolmi1/Lolmi1.home.html), *Melanconium* sp. (https://mycocosm.jgi.doe.gov/Melsp1/Melsp1.home.html), Valsa mali (https://mycocosm.jgi.doe.gov/Valma1/Valma1.home.html) (20), Valsa mali (BioSample accession number SAMN03203462), and Valsa sordida (BioSample accession number SAMN04099705).

**TABLE 1:** Assembly and annotation statistics for D. ilicicola FPH2015-502, comparison species D. helianthi, and D. ampelina

Parameter	Data for:		
	Diaporthe ilicicola (GenBank accession no. JALPVH000000000)	Diaporthe ampelina (GenBank accession no. LWAD00000000)^*a*^	Diaporthe helianthi (GenBank accession no. MAVT00000000)^*b*^
Assembly			
Genome size (bp)	65,225,202	47,325,858	63,672,038
No. of contigs	1,399	2,383	7,376
*N*_50_ (bp)	156,399	132,346	20,184
Contig *L*_50_	123	103	860
GC content (%)	44.12	53.94	43.99
Complete BUSCOs (%)^*c*^	96.3	96.3	96.4
Annotation			
No. of predicted genes	12,061	10,704	13,139
Mean gene length (bp)	1,672	1,535	1,615
Proportion of assembly covered by annotation (%)	30.92	34.71	33.32

a From Savitha et al. (21). The genome of D. ampelina is included for a robust comparison of assembly and annotation quality across *Diaporthe* spp.

b From Baroncelli et al. (9).

c The completeness (percentage of complete benchmarking universal single-copy orthologs [BUSCOs]) of the genomes was assessed using BUSCO v3.0.2 and the sordariomyceta database (release 9).

### Data availability.

The genome was deposited in DDBJ/ENA/GenBank under the accession number JALPVH000000000 (BioProject accession number PRJNA759853, BioSample accession number SAMN21205738, and SRA accession numbers SRR18821933 and SRR18821934).
